# PPARγ‐Dependent Pioglitazone Treatment Suppresses Neuroinflammation and Improves Cognition in Sepsis‐Associated Encephalopathy in Mice

**DOI:** 10.1002/cns.71103

**Published:** 2026-08-30

**Authors:** Jing Zhang, Peng Wang, Nan Li, Yan Gao

**Affiliations:** ^1^ Intensive Care Unit Yuebei People's Hospital of Shantou University Medical Shaoguan Guangdong China; ^2^ Neurosurgery The Second Affiliated Hospital of Soochow University Suzhou Jiangsu China; ^3^ Department of Emergency Medicine General Hospital of Northern Theater Command Shenyang Liaoning China

**Keywords:** apoptosis, blood–brain barrier, cognitive dysfunction, neuroinflammation, neuronal regeneration, pioglitazone, sepsis‐associated encephalopathy

## Abstract

**Objective:**

To assess whether pioglitazone (Pio) protects against sepsis‐associated encephalopathy (SAE) and to probe underlying mechanisms.

**Methods:**

Sepsis was induced in male C57BL/6 mice by caecal ligation and puncture (CLP). At 24 h post‐CLP, mice received Pio and/or the PPAR‐γ antagonist GW9662 and were followed for 14‐day survival. Cognitive and affective behaviors were evaluated using a behavioral battery. Blood–brain barrier (BBB) permeability was assessed by Evans blue extravasation, and hippocampal inflammatory and apoptotic readouts were examined by ELISA, immunoblotting, and TUNEL staining.

**Results:**

CLP reduced 14‐day survival to 21.82%, whereas Pio increased survival to 46.15%; GW9662 abolished this benefit (19.36%). Pio improved behavior consistent with reduced anxiety‐like behavior and better learning/memory. Mechanistically, Pio decreased Evans blue leakage and increased Claudin‐5, suppressed TLR4/NF‐κB–associated inflammation (lower TNF‐α, IL‐1β, and IL‐6; higher IL‐10), and reduced neuronal apoptosis; these effects were largely reversed by GW9662.

**Conclusion:**

Pioglitazone alleviates sepsis‐induced brain dysfunction and improves survival in mice in a PPAR‐γ–dependent manner.

## Graphical abstract

Pioglitazone alleviated CLP‐induced sepsis‐associated encephalopathy in mice by activating PPAR‐γ, improving survival, learning, and memory, and anxiety‐like behavior. It also protected the BBB, suppressed TLR4/NF‐κB‐associated inflammation, and reduced neuronal apoptosis, effects largely reversed by GW9662.

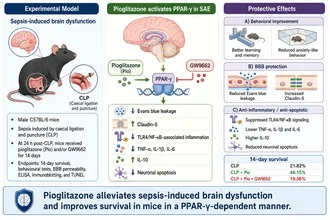

## 1 Introduction

Sepsis is defined by the Sepsis‐3 consensus as life‐threatening organ dysfunction caused by a dysregulated host response to infection. In a national survey in China, sepsis‐related deaths accounted for 67 per 100,000 total deaths in 2018—approximately tenfold higher than the estimate in 2015—underscoring a rapidly increasing mortality burden [1]. Neurological involvement is common: ~70% of patients with sepsis develop brain dysfunction spanning delirium to coma [2]. Sepsis‐associated encephalopathy (SAE) refers to a secondary, diffuse cerebral dysfunction in septic patients in the absence of direct central nervous system infection and after exclusion of other causes, and may present with delirium, agitation, memory decline, anxiety, and attentional deficits [3]. SAE is linked to higher mortality, prolonged hospitalization, and substantial socioeconomic costs, yet current sepsis guidelines provide no explicit therapeutic principles for SAE, leaving clinicians with limited targeted options [4].

Blood–brain barrier (BBB) dysfunction is increasingly implicated in SAE pathogenesis. The BBB—formed by endothelial cells with tight junctions, the basement membrane and astrocytic endfeet—selectively permits entry of essential substrates while restricting harmful factors. Disruption of astrocyte morphology and synaptic structure can increase BBB permeability [5], and impairment of Claudin‐5, a key tight‐junction protein, can raise barrier permeability and allow neurotoxic factors to access the cerebrospinal fluid and injure brain tissue [6]. BBB breakdown has been proposed as an early marker of cognitive impairment [7], and enhancing Claudin‐5 expression may preserve BBB integrity and ameliorate cognitive deficits in SAE [8]. In parallel, Toll‐like receptors (TLRs) are central pattern‐recognition receptors in innate immunity [9]; inflammatory stimuli such as lipopolysaccharide (LPS) activate TLR4 and propagate intracellular inflammatory signaling [10]. Experimental evidence indicates that suppressing the TLR4/NF‐κB axis can attenuate neuroinflammation, reduce apoptosis and support neurogenesis [7], and electroacupuncture preconditioning at Baihui (GV20) and Zusanli (ST36) has been reported to inhibit TLR4/NF‐κB signaling, limit microglia‐driven neuroinflammation, reduce hippocampal apoptosis and improve cognition in septic mice [11], positioning TLR4/NF‐κB as a key pro‐injury pathway in SAE.

Pioglitazone (Pio), a thiazolidinedione insulin sensitizer used clinically to improve insulin responsiveness while reducing hypoglycemia risk, has attracted interest for potential neuroprotective actions in disorders such as Parkinson's disease [12], traumatic brain injury [13], and Alzheimer's disease [14]. Sepsis is frequently accompanied by gastrointestinal failure and microbial translocation; pre‐treatment with Pio has been shown to reduce oxidative injury and inflammatory cytokine expression, alleviating sepsis‐associated intestinal damage and dampening systemic inflammation [15]. However, whether Pio mitigates sepsis‐induced neuropathology and cognitive impairment remains unknown. Therefore, this study examines the effects of Pio on SAE in mice and provides an initial exploration of underlying mechanisms, with emphasis on BBB integrity and inflammatory signaling.

## 2 Materials and Methods

### 2.1 Experimental Animals

A total of 233 specific pathogen‐free (SPF) male wild‐type C57BL/6 mice aged 6–8 weeks and weighing 18–22 g were obtained from Liaoning Changsheng Laboratory Animal Co. Ltd. All mice were housed in the SPF animal facility of the Trauma and War Injury Laboratory at the General Hospital of the Northern Theater Command, PLA, under controlled temperature (18°C–22°C) and humidity (55%–65%), with a 12‐h light/12‐h dark cycle and free access to food and water. Mice were allowed to acclimate to the housing environment for 3 days before the start of the experiments. No experimental intervention was performed during this period. General health status, activity, food intake, and water intake were monitored daily to ensure that the animals had adapted to the housing environment before randomization, surgery, and drug intervention. All animal procedures were approved by the institutional ethics committees of the General Hospital of the Northern Theater Command, PLA, and Dalian Medical University. Detailed information on reagents, antibodies, and equipment is provided in the Supporting Information.

### 2.2 Experimental Design, Animal Allocation, and Induction of Sepsis

**FIGURE 1:**
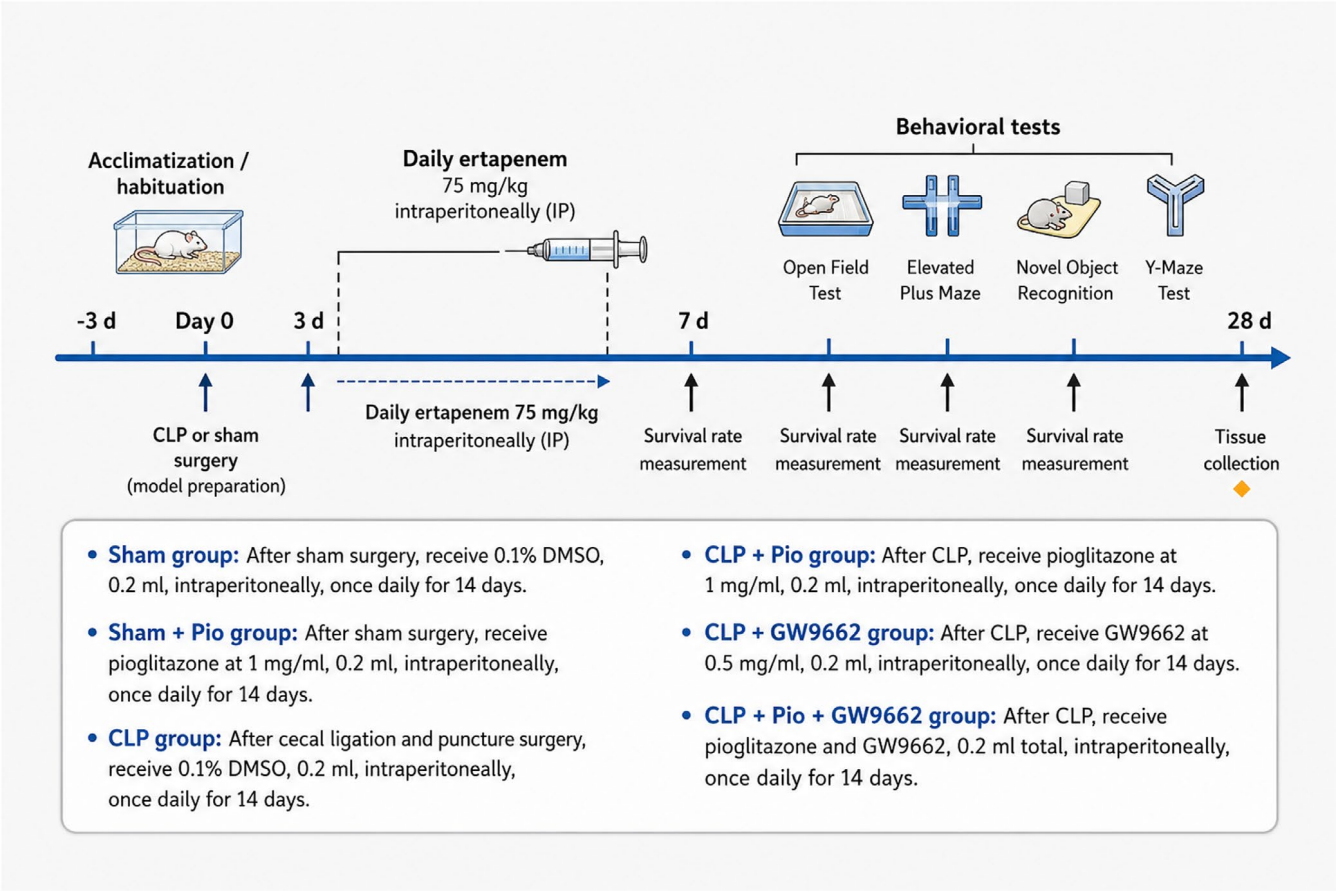
Experimental design and intervention strategy. This figure illustrates the overall experimental timeline and treatment regimens for each group. After acclimation, mice were randomly assigned to the following six groups: Sham, Sham + pioglitazone (Sham + Pio), CLP, CLP + pioglitazone (CLP + Pio), CLP + GW9662, and CLP + pioglitazone + GW9662 (CLP + Pio + GW9662). The initial numbers of mice allocated to each group were as follows: Sham, *n* = 12; Sham + Pio, *n* = 12; CLP, *n* = 55; CLP + Pio, *n* = 26; CLP + GW9662, *n* = 66; and CLP + Pio + GW9662, *n* = 62. The Sham group underwent laparotomy without cecal ligation or puncture. The Sham + Pio group received pioglitazone following sham surgery. The CLP group served as the sepsis model induced by cecal ligation and puncture. The CLP + Pio group received pioglitazone following CLP. The CLP + GW9662 group was treated with the PPARγ antagonist GW9662 after CLP. The CLP + Pio + GW9662 group received both pioglitazone and GW9662 following CLP induction. For endpoint analyses, 12 surviving mice from each group were ultimately included unless otherwise stated.

After acclimation, mice were randomly assigned using a random‐number method to six experimental groups: Sham (*n* = 12), Sham + pioglitazone (Sham + Pio; *n* = 12), CLP (*n* = 55), CLP + pioglitazone (CLP + Pio; *n* = 26), CLP + GW9662 (*n* = 66), and CLP + pioglitazone + GW9662 (CLP + Pio + GW9662; *n* = 62). These numbers represent the initial number of animals allocated to each treatment group. Because cecal ligation and puncture (CLP) induces variable mortality, a larger number of mice was initially allocated to the CLP‐treated groups to ensure that sufficient surviving animals would be available for the predefined endpoint analyses. The experimental design and workflow are summarized in Figure 1.

The survival analysis was performed using 12 mice from each group. For the sham‐operated groups, the same animals were followed for survival and subsequently used for endpoint assessment, in accordance with the principle of Reduction. For the CLP‐treated groups, animals used for survival monitoring were prospectively defined, and the remaining animals were used for endpoint experiments at the indicated time points. For endpoint analyses, 12 surviving mice from each group were ultimately included. Animals that died before the predefined endpoint or failed to meet the model criteria were not included in the endpoint analyses.

All mice were randomly assigned to experimental groups before surgery and drug intervention. Group allocation was concealed from investigators responsible for behavioral assessment, histological quantification, immunostaining analysis, Western blot quantification, ELISA measurement, and statistical analysis. The investigators performing CLP surgery and drug administration were aware of the treatment assignment because of the nature of the intervention, but they were not involved in subsequent outcome assessment. Unless otherwise stated, “*n*” represents the number of individual animals included in each analysis. The exact number of animals used for each experimental parameter is provided in the corresponding figure legends.

### 2.3 Survival Analysis

Survival analysis was performed using a predefined cohort of 12 mice from each experimental group. Survival monitoring started immediately after CLP surgery or sham operation. Mice had ad libitum access to food and water and received the corresponding interventions according to their assigned groups. Mortality was recorded once daily for 14 consecutive days after surgery. Survival rates were calculated at 3, 7, and 14 days post‐surgery, and survival curves were generated based on the 14‐day observation period. Animals that died during the observation period were included in the survival analysis but were not used for endpoint histological, biochemical, or molecular assays. For the CLP‐treated groups, the animals used for survival analysis were prospectively defined before surgery and were not included in the endpoint analyses.

### 2.4 Randomization, Allocation Concealment, and Blinding

Before surgery and drug intervention, mice were randomly assigned to experimental groups using a computer‐generated random‐number sequence by an investigator who was not involved in surgery, drug administration, outcome assessment, or data analysis. The allocation sequence was kept concealed from the investigators responsible for animal care and outcome assessment until group assignment was required. Because of the nature of the CLP procedure and drug interventions, the investigators performing surgery and drug administration were aware of the treatment allocation after group assignment. However, these investigators were not involved in behavioral assessment, histological quantification, immunostaining analysis, Western blot quantification, ELISA measurement, or statistical analysis. All outcome assessments and data analyses were performed by investigators blinded to the group allocation whenever feasible.

### 2.5 Sample Size Determination

The sample size was determined based on preliminary experiments, previous experience with the CLP‐induced sepsis model, expected mortality after CLP surgery, and the minimum number of animals required to obtain reliable endpoint measurements while following the 3Rs principles. For endpoint analyses, 12 surviving mice per group were included, which was considered sufficient to evaluate behavioral performance, histological changes, inflammatory cytokine levels, and protein expression based on our preliminary observations and commonly used sample sizes in similar experimental studies. Because CLP surgery is associated with variable mortality, especially in untreated or GW9662‐treated septic mice, a larger number of mice was initially allocated to the CLP‐related groups to ensure that at least 12 surviving animals per group would be available for the predefined endpoint analyses. In contrast, the sham‐operated groups were expected to have minimal mortality; therefore, 12 mice per sham group were considered sufficient. The initial allocation numbers were therefore adjusted according to the expected survival rate in each group while avoiding unnecessary animal use as far as possible.

### 2.6 Behavioral Assessment

To evaluate locomotor activity, anxiety‐like behavior, and cognitive function, mice underwent a battery of behavioral tests, including the open field test, elevated plus maze, novel object recognition test, and Y‐maze task. Mice were transferred to the behavioral testing room 24 h before testing for habituation. After each test, all apparatus surfaces were cleaned with 75% ethanol to eliminate odor cues. Detailed testing procedures and calculation formulas are provided in the Supporting Information [16, 17, 18, 19].

### 2.7 Quantification of Inflammatory Cytokines in Hippocampal Tissue

Frozen hippocampal samples were thawed on ice and homogenized in lysis buffer. After centrifugation at 12,000 r.p.m. for 20 min at 4°C, the supernatant was collected and total protein concentration was measured by BCA assay. Samples were diluted 1:20, and the levels of TNF‐α, IL‐1β, IL‐6, and IL‐10 were determined using ELISA kits according to the manufacturers' instructions. Cytokine concentrations were expressed as pg/mL.

### 2.8 Western Blot Analysis

Hippocampal tissue lysates were prepared as described above. Protein concentration was quantified by BCA assay, and samples were normalized to 2 μg μL^−1^. Equal amounts of protein were separated by 10% SDS–PAGE and transferred onto membranes. Membranes were blocked in skim milk and incubated with primary antibodies against TLR4, NF‐κB, Claudin‐5, Bax, Bcl‐2, caspase‐3, and β‐actin, followed by incubation with the appropriate HRP‐conjugated secondary antibodies. Protein bands were visualized using ECL substrate and quantified using the Tanon imaging system. Antibody information and dilution ratios are listed in the Supporting Information. Detailed information on the antibody types and dilution ratios used for western blot experiments is provided in Supplementary Table S1.

### 2.9 Immunofluorescence Staining

Blood–brain barrier permeability was evaluated by Evans blue extravasation. Neuronal apoptosis was assessed by TUNEL staining, and neuronal regeneration‐related changes were examined by BrdU/NeuN double immunofluorescence. Detailed procedures for tissue perfusion, fixation, sectioning, staining, and image acquisition are described in the Supporting Information.

### 2.10 Statistical Analysis

ImageJ and Image‐Pro Plus 6.0 were used to quantify western blot band intensities and immunofluorescence signals, respectively. All quantitative data are presented as mean ± SEM. Statistical analyses were performed using GraphPad Prism 7. For comparisons among multiple groups, one‐way analysis of variance (one‐way ANOVA) followed by Fisher's least significant difference (LSD) test was used. A two‐sided *p* value < 0.05 was considered statistically significant.

## 3 Results

### 3.1 Pioglitazone Significantly Improved 14‐Day Survival in Septic Mice

**FIGURE 2:**
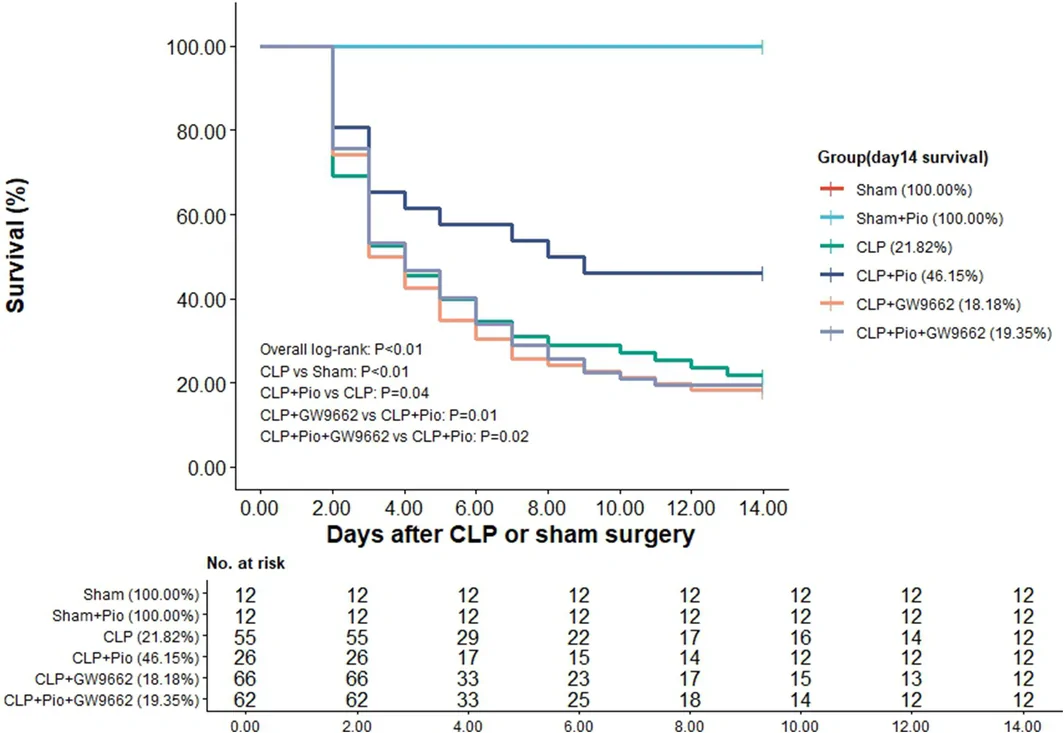
Kaplan–Meier survival curves of mice following CLP or sham surgery. Mice in the Sham (*n* = 12), Sham + Pio (*n* = 12), CLP (*n* = 55), CLP + Pio (*n* = 26), CLP + GW9662 (*n* = 66), and CLP + Pio + GW9662 (*n* = 62) groups were monitored for survival for 14 consecutive days. The respective survival rates on day 14 were 100.00%, 100.00%, 21.82%, 46.15%, 18.18%, and 19.35%. The x‐axis represents days after cecal ligation and puncture (CLP) or sham surgery, and the y‐axis represents survival probability (%). Numbers at risk are presented below the survival curves at 2‐day intervals. Differences in survival distributions were assessed using the log‐rank test. The overall difference among the six groups was statistically significant (*p* < 0.01). Pairwise log‐rank comparisons revealed significant differences between the CLP and Sham groups (*p* < 0.01), the CLP + Pio and CLP groups (*p* = 0.04), the CLP + GW9662 and CLP + Pio groups (*p* = 0.01), and the CLP + Pio + GW9662 and CLP + Pio groups (*p* = 0.02).

As shown in Figure 2, all mice in the Sham and Sham + Pio groups survived throughout the 14‐day observation period, indicating that pioglitazone itself did not adversely affect survival under physiological conditions. In contrast, CLP markedly reduced survival, and most deaths occurred during the early post‐operative phase, particularly within the first 7 days, after which the survival curve gradually plateaued. Pioglitazone treatment improved survival in septic mice, increasing the 14‐day survival rate from 21.82% in the CLP group to 46.15% in the CLP + Pio group. Importantly, this survival benefit was lost after co‐administration of the PPAR‐γ antagonist GW9662, as the 14‐day survival rate in the CLP + Pio + GW9662 group decreased to 19.36%, a level comparable to that of the CLP and CLP + GW9662 groups. No further deaths were observed after day 14. Taken together, these findings indicate that pioglitazone confers a significant survival advantage in septic mice and that this protective effect is largely dependent on PPAR‐γ activation.

### 3.2 Pioglitazone Alleviated Sepsis‐Induced Behavioral and Cognitive Impairment

To assess whether pioglitazone ameliorated sepsis‐associated encephalopathy, mice were subjected to a battery of behavioral tests during postoperative days 21–28, including the open‐field test, elevated plus maze, novel object recognition task, and Y‐maze. Across all paradigms, Sham and Sham + Pio mice performed similarly, suggesting that pioglitazone did not significantly alter locomotor activity, anxiety‐related behavior, or cognitive performance in healthy animals. In contrast, CLP mice consistently exhibited reduced spontaneous activity, diminished exploratory behavior, enhanced anxiety‐like behavior, and impaired learning and memory. These behavioral abnormalities were markedly improved by pioglitazone treatment but were substantially attenuated or abolished when GW9662 was co‐administered, supporting the conclusion that the neurobehavioral benefits of pioglitazone are mediated, at least in part, through PPAR‐γ.

#### 3.2.1 Pioglitazone Improved Locomotor Activity, Exploratory Behavior, and Anxiety‐Like Behavior in Septic Mice

**FIGURE 3:**
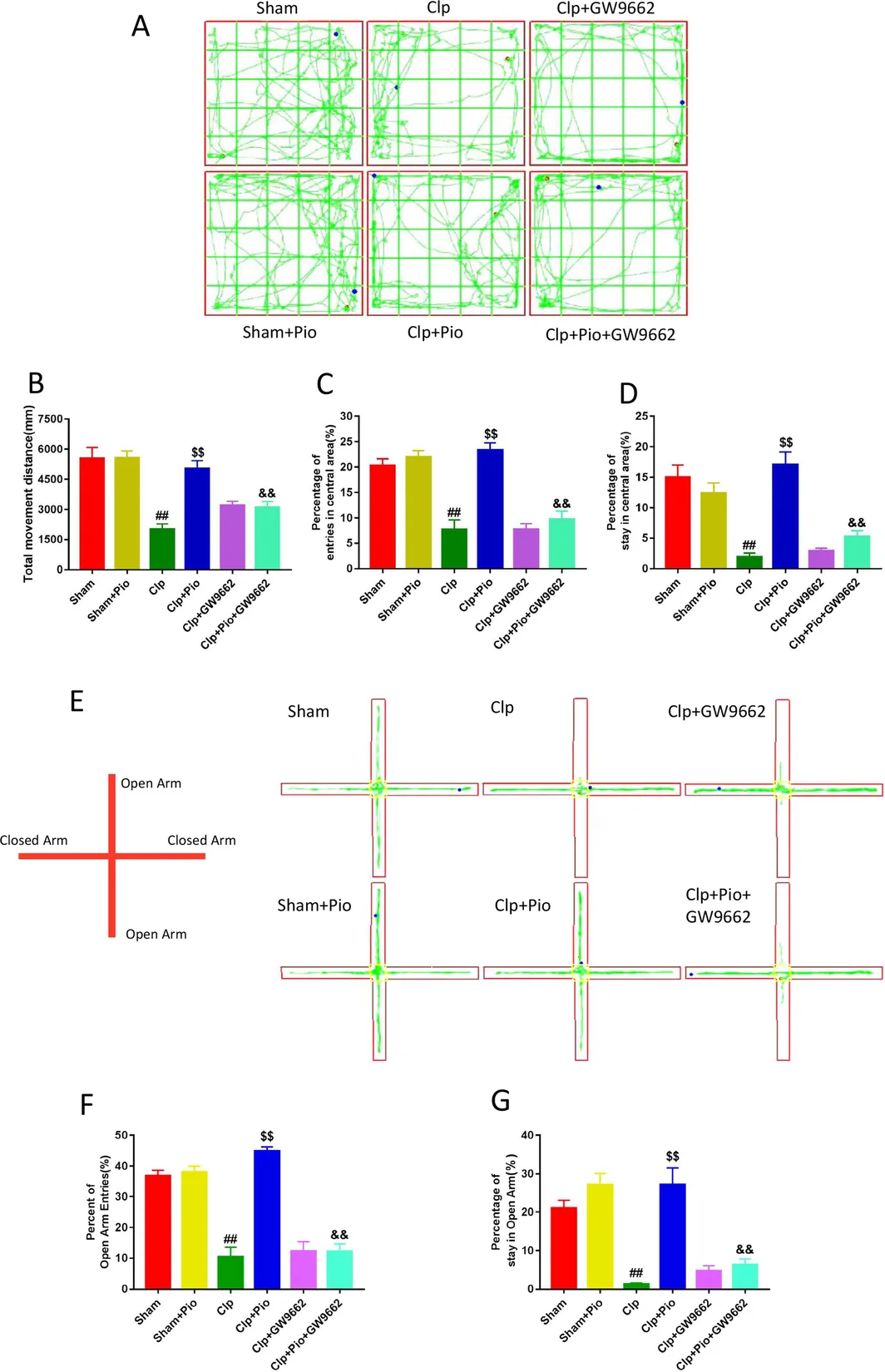
Effects of pioglitazone and GW9662 on open‐field and elevated plus‐maze performance. General locomotor activity was assessed using the open‐field test. Data are presented as mean ± SEM. (A) Representative locomotor trajectories for each group in the open‐field arena. (B) Total distance traveled (mm; *n* = 12 mice per group). (C) Percentage of entries into the center zone (%; *n* = 12 mice per group). (D) Percentage of time spent immobile in the center zone (%; *n* = 12 mice per group). Anxiety‐like behavior was evaluated using the elevated plus maze. (E) Representative movement trajectories for each group in the elevated plus maze. (F) Percentage of entries into the open arms (%; *n* = 12 mice per group). (G) Percentage of time spent in the open arms (%; *n* = 12 mice per group). Unless otherwise stated, *n* represents the number of individual animals included in each analysis. Statistical significance is indicated as follows: ##*p* < 0.01 versus the Sham group; $$*p* < 0.01 versus the CLP group; &&*p* < 0.01 versus the CLP + Pio group.

Representative open‐field trajectories and quantitative analyses are shown in Figure 3A–C. Sham and Sham + Pio mice explored the arena extensively and entered the center zone frequently, whereas CLP mice displayed marked hypoactivity, reduced movement range, and limited center exploration. These findings indicate that sepsis profoundly impaired general locomotor and exploratory behavior. Pioglitazone treatment substantially restored these behavioral indices, as reflected by increased total distance traveled, a higher proportion of center entries, and greater center exploration. By contrast, co‐treatment with GW9662 significantly diminished these improvements. Thus, pioglitazone effectively ameliorated the reduction in locomotor activity and exploratory behavior induced by sepsis.

A similar pattern was observed in the elevated plus maze, shown in Figure 3E–G. Sham and Sham + Pio mice frequently entered and remained in the open arms, indicating a normal exploratory response and low anxiety‐like behavior. In contrast, CLP mice showed marked avoidance of the open arms, with significant reductions in both open‐arm entry percentage and time spent in the open arms, consistent with an anxiety‐like phenotype. Pioglitazone significantly increased open‐arm exploration in septic mice, indicating an anxiolytic‐like effect. However, this effect was abolished by GW9662, which reduced both the frequency and duration of open‐arm exploration to levels comparable with those seen in untreated CLP mice. These data demonstrate that pioglitazone alleviates sepsis‐induced anxiety‐like behavior in a PPAR‐γ‐dependent manner.

#### 3.2.2 Pioglitazone Improved Learning and Memory Performance in Septic Mice

**FIGURE 4:**
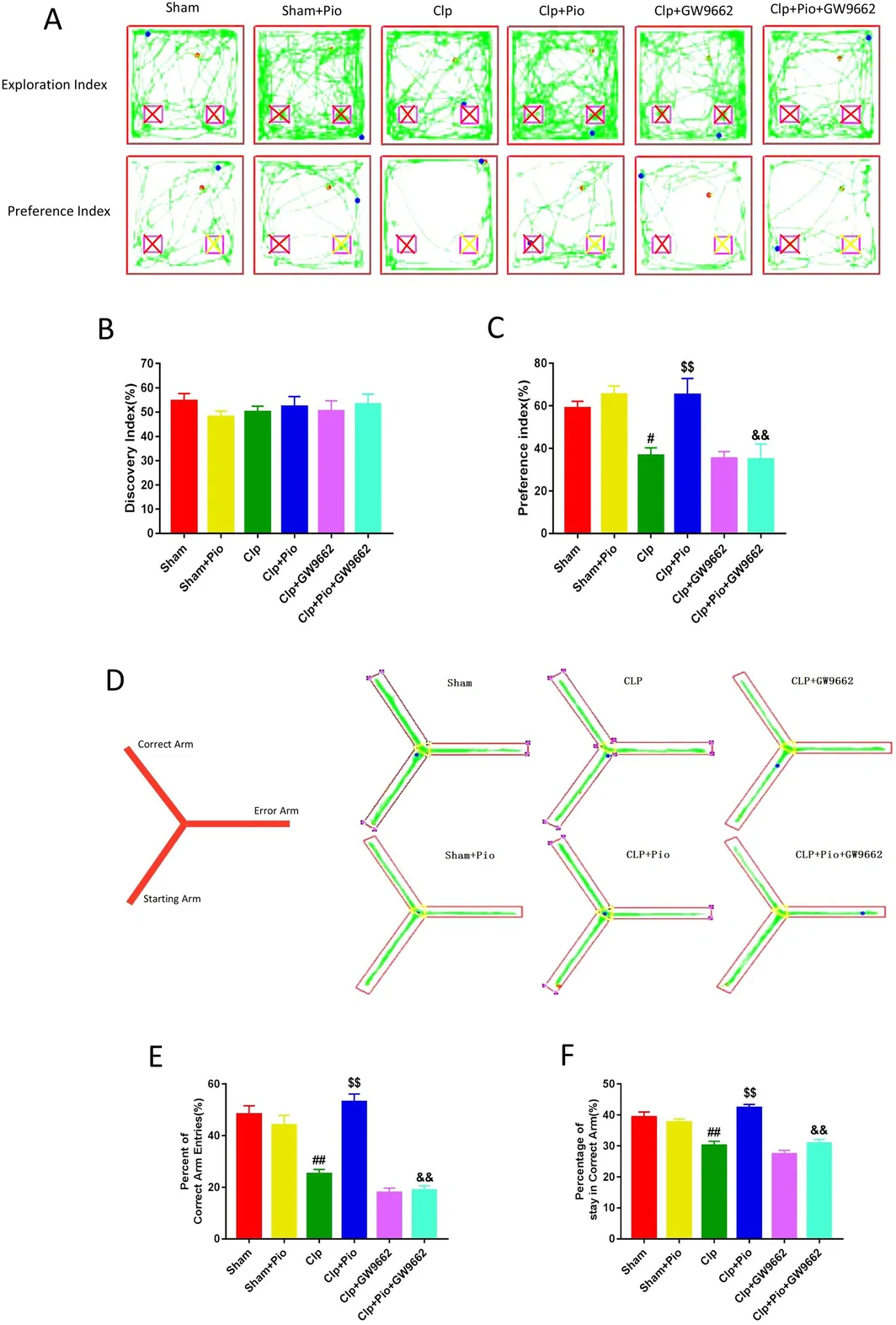
Effects of pioglitazone and GW9662 on novel object recognition and Y‐maze performance. Novel object recognition was used to evaluate exploratory behavior and recognition memory across groups. Data are presented as mean ± SEM. (A) Representative movement trajectories during the novel object recognition task. (B) Exploration index (%; *n* = 12 mice per group). (C) Preference index (%; *n* = 12 mice per group). The Y‐maze test was used to compare spatial learning and memory among groups. (D) Representative movement trajectories in the Y‐maze. (E) Percentage of entries into the correct arm (%; *n* = 12 mice per group). (F) Percentage of time spent in the correct arm (%; *n* = 12 mice per group). Unless otherwise stated, *n* represents the number of individual animals included in each analysis. Statistical significance is indicated as follows: #*p* < 0.05 and ##*p* < 0.01 versus the Sham group; $$*p* < 0.01 versus the CLP group; &&*p* < 0.01 versus the CLP + Pio group.

The novel object recognition task further showed that pioglitazone improved recognition memory in septic mice. As shown in Figure 4A–C, the exploration index during the familiarization phase did not differ substantially among groups, indicating comparable baseline object exploration. However, during the test phase, Sham and Sham + Pio mice displayed a clear preference for the novel object, whereas CLP mice exhibited a markedly reduced preference index, reflecting impaired recognition memory. Pioglitazone significantly restored the preference for the novel object in septic mice, while GW9662 reversed this improvement. These results indicate that pioglitazone improves sepsis‐induced recognition memory deficits.

Consistent findings were obtained in the Y‐maze task, shown in Figure 4D–F. Sham and Sham + Pio mice preferentially entered the correct arm and spent more time there, reflecting intact spatial learning and memory. By contrast, CLP mice displayed disorganized exploration, reduced correct‐arm entry percentage, and decreased dwell time in the correct arm, indicating impaired spatial memory performance. Pioglitazone significantly increased both correct‐arm entry percentage and dwell time in septic mice, whereas co‐administration of GW9662 attenuated these improvements. Taken together, the behavioral data from the open‐field test, elevated plus maze, novel object recognition, and Y‐maze consistently demonstrate that CLP‐induced sepsis causes substantial neurobehavioral and cognitive dysfunction, and that pioglitazone markedly ameliorates these deficits through a mechanism involving PPAR‐γ.

### 3.3 Pioglitazone Preserved Blood–Brain Barrier Integrity and Reduced Hippocampal Apoptosis in Septic Mice

**FIGURE 5:**
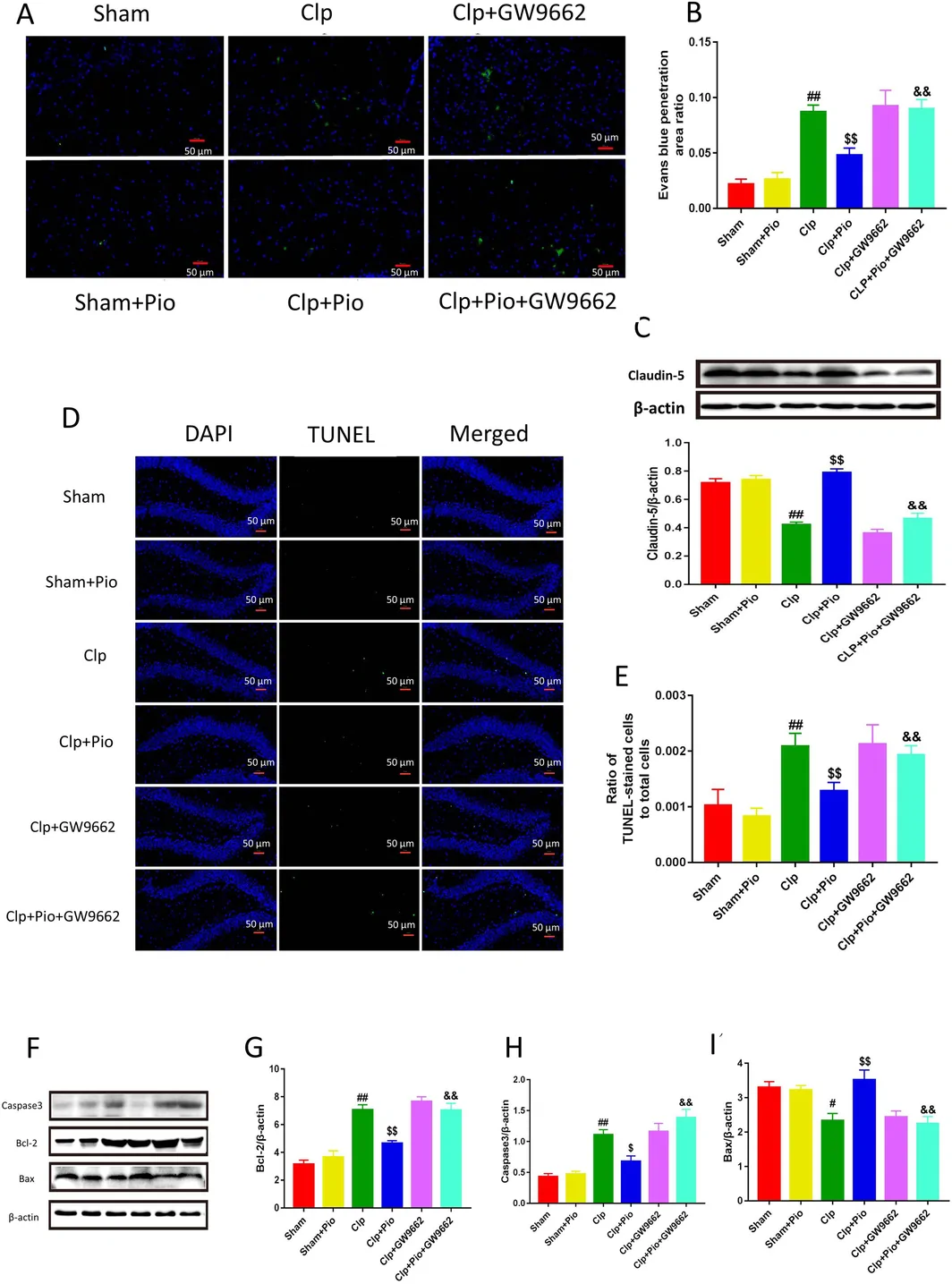
Pioglitazone and GW9662 modulate hippocampal blood–brain barrier permeability, Claudin‐5 expression, and neuronal apoptosis in mice with sepsis‐associated encephalopathy. Blood–brain barrier integrity was evaluated by quantifying Evans blue extravasation following tail‐vein injection and by measuring Claudin‐5 protein expression. Data are presented as mean ± SEM. (A) Representative fluorescence micrographs of Evans blue leakage in the hippocampus. (B) Percentage area of Evans blue extravasation (*n* = 12 mice per group). (C) Claudin‐5/β‐actin ratio in the hippocampus, as determined by western blotting (*n* = 12 mice per group). Cell apoptosis at day 28 was assessed by TUNEL staining and compared across groups. (D) Representative TUNEL staining images of the hippocampal dentate gyrus after pioglitazone and GW9662 treatment. Nuclei were counterstained with DAPI in blue, and apoptotic cells were TUNEL‐positive in green. (E) Quantification of apoptosis, expressed as the ratio of TUNEL‐positive cells to total cells in the hippocampal dentate gyrus (*n* = 12 mice per group). Apoptosis‐related proteins, including Caspase‐3, Bcl‐2, and Bax, in hippocampal tissue were quantified by western blotting. (F) Representative immunoblot bands of Caspase‐3, Bcl‐2, and Bax in hippocampal lysates. (G) Caspase‐3/β‐actin ratio in the hippocampus (*n* = 12 mice per group). (H) Bcl‐2/β‐actin ratio in the hippocampus (*n* = 12 mice per group). (I) Bax/β‐actin ratio in the hippocampus (*n* = 12 mice per group). For histological quantification, multiple fields or sections were analyzed from each animal, and the individual animal was considered the biological unit for statistical analysis. Unless otherwise stated, *n* represents the number of individual animals included in each analysis. Statistical significance is indicated as follows: #*p* < 0.05 and ##*p* < 0.01 versus the Sham group; $*p* < 0.05 and $$*p* < 0.01 versus the CLP group; &&*p* < 0.01 versus the CLP + Pio group.

To determine whether pioglitazone protected blood–brain barrier integrity after sepsis, Evans blue extravasation was examined in hippocampal tissue. Representative fluorescence images and quantitative analysis are shown in Figure 5A,B. Only minimal Evans blue leakage was detected in the hippocampi of Sham and Sham + Pio mice, consistent with an intact blood–brain barrier under basal conditions. In contrast, CLP mice exhibited prominent patchy Evans blue extravasation, indicating marked blood–brain barrier disruption. Pioglitazone treatment markedly reduced the Evans blue‐positive area in the hippocampus, whereas GW9662 abolished this protective effect and restored extensive leakage. These findings indicate that pioglitazone significantly attenuates sepsis‐induced blood–brain barrier damage.

The Evans blue findings were further supported by analysis of the tight‐junction protein Claudin‐5, shown in Figure 5C. Claudin‐5 expression did not differ between the Sham and Sham + Pio groups, but was markedly reduced in the CLP group, consistent with blood–brain barrier breakdown. Pioglitazone significantly restored Claudin‐5 expression in septic mice, whereas GW9662 attenuated this recovery. Together, the reduced Evans blue leakage and restored Claudin‐5 abundance indicate that pioglitazone preserves blood–brain barrier integrity in the septic hippocampus in a PPAR‐γ‐dependent manner.

We next examined whether pioglitazone reduced neuronal apoptosis in the hippocampus. Representative TUNEL staining and quantitative analyses are shown in Figure 5D,E. Very few TUNEL‐positive cells were observed in the dentate gyrus of Sham and Sham + Pio mice, whereas CLP mice exhibited a marked increase in apoptotic cells, indicating substantial neuronal injury after sepsis. Pioglitazone treatment significantly reduced the number of TUNEL‐positive cells in septic mice, while co‐treatment with GW9662 abolished this anti‐apoptotic effect. Thus, pioglitazone attenuated hippocampal neuronal apoptosis induced by CLP.

The anti‐apoptotic effect of pioglitazone was further supported by western blot analysis of apoptosis‐related proteins, shown in Figure 5F–I. Compared with Sham controls, CLP markedly altered the expression of Caspase‐3, Bcl‐2, and Bax, indicating activation of apoptosis‐related signaling in the hippocampus. Pioglitazone treatment shifted the expression of these proteins in a direction consistent with reduced apoptosis, whereas GW9662 reversed these changes. Taken together with the TUNEL data, these findings indicate that pioglitazone suppresses hippocampal apoptosis in septic mice and that this protective effect is largely dependent on PPAR‐γ signaling.

### 3.4 Pioglitazone Attenuated Hippocampal Inflammation by Suppressing the TLR4–NF‐κB Signaling Pathway

**FIGURE 6:**
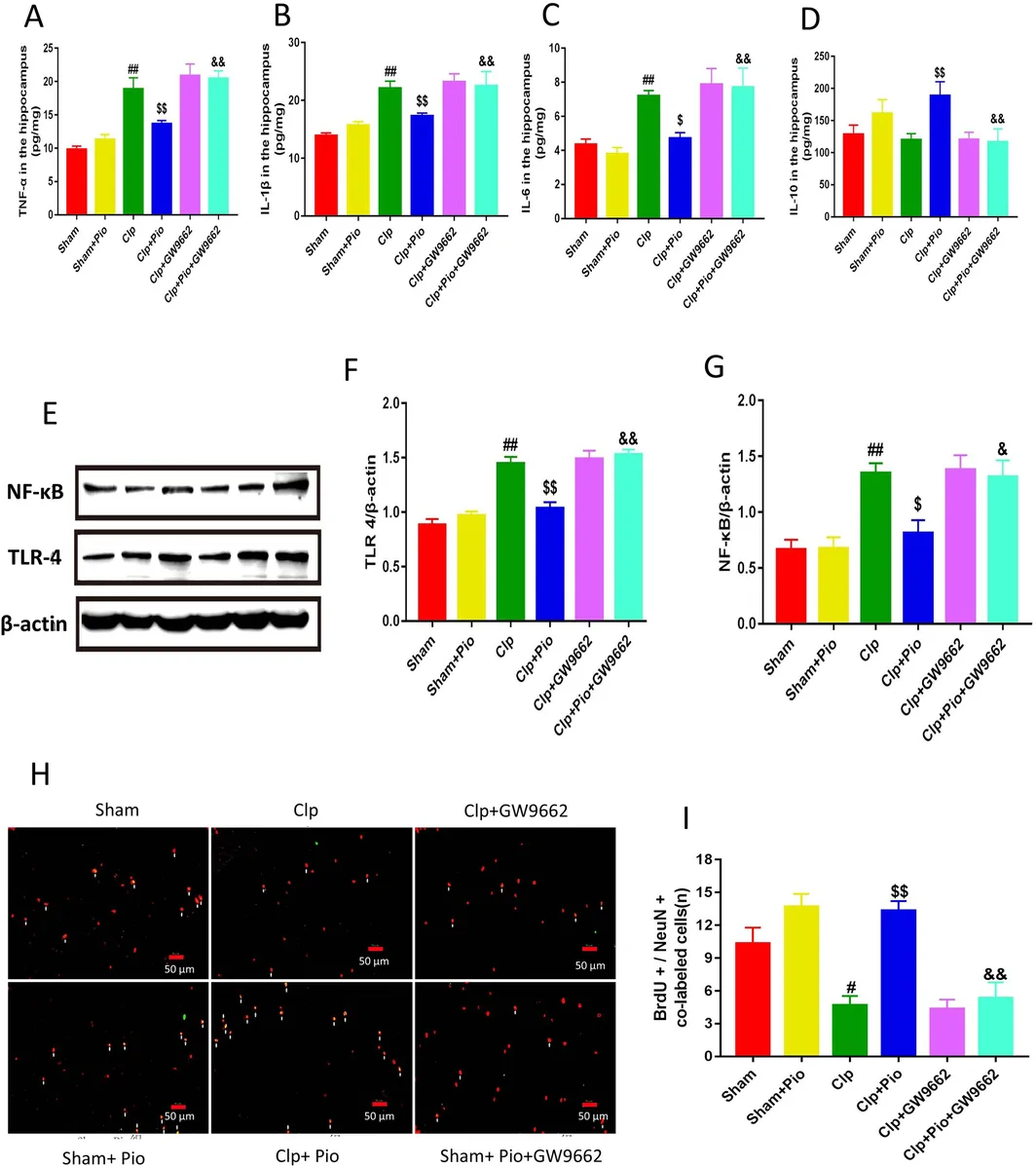
Pioglitazone and GW9662 modulate hippocampal inflammation, TLR4–NF‐κB signaling, and NeuN+/BrdU+ double‐positive cells in septic mice. (A–D) Hippocampal levels of pro‐inflammatory and anti‐inflammatory cytokines in mice from different experimental groups. Data are presented as mean ± SEM. (A) TNF‐α level in hippocampal tissue (pg/mg; *n* = 12 mice per group). (B) IL‐1β level in hippocampal tissue (pg/mg; *n* = 12 mice per group). (C) IL‐6 level in hippocampal tissue (pg/mg; *n* = 12 mice per group). (D) IL‐10 level in hippocampal tissue (pg/mg; *n* = 12 mice per group). (E–G) Representative immunoblots and quantitative analysis of hippocampal TLR4 and NF‐κB protein expression after pioglitazone and/or GW9662 treatment. TLR4 and NF‐κB protein levels were assessed by western blotting and normalized to β‐actin. (E) Representative western blot bands for TLR4, NF‐κB, and β‐actin in hippocampal lysates. (F) Quantification of relative TLR4 protein expression (TLR4/β‐actin; *n* = 12 mice per group). (G) Quantification of relative NF‐κB protein expression (NF‐κB/β‐actin; *n* = 12 mice per group). (H, I) NeuN+/BrdU+ double‐positive cells in the hippocampal region of mice with sepsis‐associated encephalopathy after pioglitazone and/or GW9662 treatment. At day28, hippocampal sections were co‐stained with NeuN and BrdU. (H) Representative fluorescence images of NeuN+/BrdU+ double‐labeled cells in the hippocampus. BrdU+ cells are shown in green, and NeuN+ neurons are shown in red. (I) Quantification of NeuN+/BrdU+ double‐positive cells (*n* = 3 mice per group). For ELISA and western blotting, *n* represents the number of individual animals included in each analysis. For immunofluorescence analysis, multiple sections or microscopic fields were analyzed from each animal, and the individual animal was considered the biological unit for statistical analysis. Statistical significance is indicated as follows: #*p* < 0.05 and ##*p* < 0.01 versus the Sham group; $*p* < 0.05 and $$*p* < 0.01 versus the CLP group; and &*p* < 0.05 and &&*p* < 0.01 versus the CLP + Pio group.

To determine whether pioglitazone modulated the hippocampal inflammatory response after sepsis, we first quantified cytokine levels by ELISA. As shown in Figure 6A–D, CLP markedly increased the concentrations of the pro‐inflammatory cytokines TNF‐α, IL‐1β, and IL‐6 in hippocampal tissue, whereas pioglitazone significantly reduced all three cytokines. In parallel, pioglitazone increased the anti‐inflammatory cytokine IL‐10. These anti‐inflammatory effects were largely abolished by GW9662, while GW9662 alone did not significantly alter cytokine levels compared with the CLP group. Thus, pioglitazone shifted the hippocampal inflammatory milieu from a strongly pro‐inflammatory state toward a more balanced, anti‐inflammatory profile.

We next examined the TLR4–NF‐κB signaling pathway, which is a key upstream regulator of inflammatory amplification during sepsis. Representative western blots and densitometric analyses are shown in Figure 6E–G. Relative to the Sham group, CLP significantly increased hippocampal TLR4 and NF‐κB protein expression, indicating activation of this inflammatory cascade. Pioglitazone markedly suppressed both TLR4 and NF‐κB expression in septic mice, whereas co‐administration of GW9662 reversed these inhibitory effects. These findings strongly suggest that pioglitazone attenuates hippocampal inflammation by suppressing the TLR4–NF‐κB signaling axis via PPAR‐γ activation, thereby reducing downstream pro‐inflammatory cytokine production and mitigating central nervous system inflammation during sepsis.

### 3.5 Pioglitazone Increased NeuN ^+^/BrdU ^+^ Double‐Positive Cells in the Hippocampus

Finally, to assess whether pioglitazone influenced neuronal regeneration‐related changes after sepsis, NeuN/BrdU double immunofluorescence staining was performed. As shown in Figure 6H,I, Sham and Sham + Pio mice exhibited comparable numbers of NeuN^+^/BrdU^+^ double‐positive cells in the hippocampal region, indicating that pioglitazone did not alter this parameter under basal conditions. In contrast, CLP markedly reduced the abundance of NeuN^+^/BrdU^+^ cells, suggesting that sepsis impaired hippocampal neuronal renewal or regeneration‐related processes. Pioglitazone treatment significantly increased the number of NeuN^+^/BrdU^+^ double‐positive cells compared with the CLP group, whereas GW9662 attenuated this increase. These findings suggest that, in addition to its anti‐inflammatory, blood–brain barrier‐protective, and anti‐apoptotic effects, pioglitazone may also promote neuronal recovery‐related changes in the hippocampus after sepsis, at least in part through activation of PPAR‐γ.

## 4 Discussion

Among the currently available experimental approaches for modeling sepsis, exogenous lipopolysaccharide administration, live bacterial inoculation, and cecal ligation and puncture (CLP) are the most widely used. Of these, CLP is generally regarded as the most clinically relevant model because it induces polymicrobial infection through disruption of the intestinal barrier and more closely recapitulates the dynamic pathophysiology of human sepsis. In addition, CLP is highly reproducible and has therefore been widely adopted as a standard experimental model of severe sepsis [20, 21]. Previous studies have reported that the 7‐day survival rate of male mice subjected to severe CLP is approximately 20%–25% [22, 23]. In the present study, the survival profile of the untreated CLP group was consistent with these reports, supporting the successful establishment of a severe sepsis model. Against this background, our data demonstrate that pioglitazone (Pio), a PPAR‐γ agonist, significantly improved survival in septic mice, whereas this effect was abolished by the PPAR‐γ antagonist GW9662. These findings extend previous observations that Pio enhances bacterial clearance and survival in sepsis [24], attenuates sepsis‐induced acute lung injury [25], and suppresses NF‐κB‐dependent inflammatory responses in systemic infection [26]. Collectively, our results support the view that Pio exerts a meaningful protective effect in severe sepsis and suggest that its benefits extend beyond peripheral organ protection to include the central nervous system.

Increasing evidence indicates that Pio also has neuroprotective properties. Previous studies have shown that Pio activates hippocampal PPAR‐γ, alleviates anxiety‐related behavior, and reduces withdrawal‐associated symptoms [27]. In addition, Pio has been reported to attenuate neuroinflammation and improve cognitive dysfunction in Alzheimer's disease [14], and to reduce blood–brain barrier damage and neuronal apoptosis in epilepsy through activation of PPAR‐γ [28]. Hontecillas et al. further demonstrated that increased PPAR‐γ activity in macrophages suppresses TLR4 expression and limits the release of inflammatory mediators [29]. These observations provided the mechanistic rationale for examining whether Pio could ameliorate sepsis‐associated encephalopathy (SAE), a diffuse brain dysfunction secondary to sepsis that is strongly associated with delirium, affective symptoms, and persistent cognitive impairment [30]. Behavioral studies have shown that cognitive dysfunction after CLP may emerge as early as 10 days, persist for several weeks, and gradually recover over time [31, 32]. In the present study, we therefore assessed neurobehavioral outcomes during the subacute phase after CLP using complementary assays of locomotion, anxiety‐like behavior, recognition memory, and spatial learning. CLP produced a consistent behavioral phenotype characterized by reduced exploratory activity, increased anxiety‐like behavior, and impaired learning and memory, whereas Pio substantially improved each of these abnormalities. Importantly, GW9662 reversed these behavioral benefits, strongly indicating that the protective effect of Pio on cognitive dysfunction is mediated, at least in part, through activation of PPAR‐γ. Thus, beyond improving survival, Pio appears to preserve higher‐order neurological function in septic mice.

The behavioral benefit observed in this study is biologically plausible in light of the known pathological mechanisms of SAE. Among the mechanisms currently implicated, blood–brain barrier (BBB) dysfunction and neuroinflammation are particularly important because they amplify one another and together drive neuronal injury and persistent cognitive decline. The BBB is a highly specialized interface formed by endothelial cells, tight junction proteins, the basement membrane, and astrocytic end‐feet, and its disruption permits circulating inflammatory mediators and toxic molecules to access the brain parenchyma [33]. This process has been implicated not only in the acute phase of SAE but also in the maintenance of long‐term cognitive impairment after sepsis [33]. In parallel, apoptosis is increasingly recognized as an important downstream effector of septic brain injury. Previous studies have shown that CAPON can regulate Caspase‐3 expression and promote apoptosis in the hippocampus [34], while LPS stimulation in microglia induces both inflammatory mediator release and apoptotic activation [35]. In SAE models, neuroinflammation has likewise been linked to enhanced apoptosis [36]. Against this background, it is notable that Pio has been shown to reduce apoptosis in other injury settings, including intervertebral disc degeneration [37], and to attenuate β‐amyloid‐associated injury in Alzheimer's disease [38]. In our study, CLP increased neuronal apoptosis in the hippocampus, accompanied by higher Caspase‐3 and Bax expression and lower Bcl‐2 expression, whereas Pio markedly reversed these changes. Moreover, the protective effect of Pio on apoptosis was lost after pharmacological inhibition of PPAR‐γ. Together with the observed reduction in Evans blue extravasation and restoration of Claudin‐5 expression, these findings support a model in which Pio preserves BBB integrity and suppresses hippocampal apoptosis, thereby interrupting a central pathological axis of SAE.

Our data further identify suppression of the TLR4/NF‐κB pathway as a likely mechanism linking PPAR‐γ activation to reduced neuroinflammation. LPS and other septic mediators can trigger central inflammatory responses through at least two convergent routes: by directly engaging CD14/TLR4 signaling in microglia and by activating astrocytes, both of which promote the release of inflammatory mediators and amplify neuroinflammation [39]. Previous work has shown that activation of the TLR4/NF‐κB pathway contributes to neuronal apoptosis and cognitive impairment in SAE [40]. Pio has been reported in other disease models to suppress this inflammatory axis, including in melanoma‐bearing mice [41] and in steatohepatitis, where PPAR‐γ activation inhibits TLR4 signaling and reduces inflammatory cytokine production [42]. However, whether this mechanism operates in SAE has remained insufficiently defined. In the present study, septic mice exhibited marked upregulation of hippocampal TLR4 and NF‐κB, together with elevated TNF‐α, IL‐1β, and IL‐6, indicating robust activation of inflammatory signaling in the brain. Pio significantly reduced TLR4 and NF‐κB expression, lowered pro‐inflammatory cytokine levels, and increased IL‐10, whereas GW9662 largely abolished these effects. These results strongly suggest that Pio suppresses hippocampal neuroinflammation in SAE by activating PPAR‐γ and thereby restraining the TLR4/NF‐κB cascade. From a mechanistic perspective, this pathway provides a coherent framework linking Pio treatment to preserved BBB function, reduced apoptosis, and improved behavioral outcomes.

Another noteworthy finding of this study is that Pio increased the abundance of NeuN^+^/BrdU^+^ double‐positive cells in the hippocampus. Although the interpretation of such staining should remain cautious, this result is consistent with enhanced neuronal regeneration‐related activity or a more favorable neurogenic microenvironment after sepsis. Interest in adult hippocampal neurogenesis has increased substantially in recent years. A 2018 Nature study suggested that neural progenitor cells may persist in the human dentate gyrus across the lifespan and that their activity is highly dependent on the surrounding microenvironment [35]. Additional studies have indicated that progenitor cells in the subventricular zone and subgranular zone may migrate toward injured brain regions during regeneration [43]. Age‐related declines in hippocampal neurogenesis have also been linked to reduced cognitive performance, and modulation of regulators such as Tet2 has been shown to partially reverse this process [44]. In parallel, Pio has been reported to protect neuronal function after peripheral nerve injury [45] and to promote neuronal regeneration and improve functional recovery in stroke models by suppressing inflammation and apoptosis [46]. Our findings are consistent with this broader literature and suggest that Pio may not only limit ongoing injury in SAE but also facilitate repair‐related processes within the hippocampus. Such a dual action—reducing damage while promoting recovery—may help explain the relatively broad behavioral benefit observed in the present study.

Several limitations should be acknowledged when interpreting these findings. First, although our data strongly support involvement of the PPAR‐γ/TLR4/NF‐κB axis, the downstream molecular network was not dissected in greater detail. Future studies incorporating transcriptomic profiling, cell‐type‐specific analyses, and targeted pathway perturbation would help to clarify how PPAR‐γ coordinates inflammatory, apoptotic, and reparative responses in SAE. Second, our analysis focused primarily on the hippocampus, given its central role in memory and affective regulation; however, SAE is a diffuse brain disorder, and additional work is needed to determine whether similar protective effects occur in other vulnerable regions, such as the cortex, amygdala, or white matter tracts. Third, although GW9662 provided useful pharmacological evidence for the role of PPAR‐γ, genetic approaches would strengthen causal inference and better define cell‐specific mechanisms. Finally, the present study was conducted in a single experimental model and therefore requires validation in additional preclinical settings before translation can be considered. Despite these limitations, the overall pattern of findings was internally consistent across survival, behavioral, molecular, and histological outcomes, supporting the robustness of the central conclusion.

A limitation of the present study is that p‐PPAR‐γ and total PPAR‐γ expression were not directly measured by Western blotting. Although the attenuation of pioglitazone‐mediated protection by GW9662 supports the involvement of a PPAR‐γ‐dependent mechanism, direct assessment of p‐PPAR‐γ, total PPAR‐γ, and the p‐PPAR‐γ/PPAR‐γ ratio would provide stronger evidence for PPAR‐γ activation. Future studies with sufficient matched tissue samples and validated antibodies are needed to further confirm this mechanism. Another limitation is that NeuN+/BrdU+ double‐positive cells were used to evaluate a neurogenesis‐associated response, but additional neurogenesis‐related markers, such as DCX, Nestin, and Sox2, were not assessed. Therefore, the increase in NeuN+/BrdU+ double‐positive cells should not be interpreted as definitive evidence of mature neuronal regeneration. Future studies should include multiple neurogenesis markers and lineage‐tracing approaches to better distinguish neuronal regeneration from other cellular proliferative processes.

In conclusion, the present study demonstrates that Pio exerts significant neuroprotective effects in SAE. By activating PPAR‐γ, Pio suppresses TLR4/NF‐κB‐mediated neuroinflammation, preserves BBB integrity, attenuates hippocampal neuronal apoptosis, promotes regeneration‐related changes, and ultimately improves cognitive dysfunction in septic mice. These findings provide experimental support for Pio as a potential therapeutic strategy for SAE and warrant further investigation into its translational potential.

## 5 Conclusion

In conclusion, pioglitazone exerts significant neuroprotective effects in sepsis‐associated encephalopathy by suppressing neuroinflammation, preserving blood–brain barrier integrity, reducing hippocampal neuronal apoptosis, promoting neuronal proliferation, and improving cognitive dysfunction. PPAR‐γ appears to be a key molecular mediator of these protective effects.

## Author Contributions

Jing Zhang: conceptualization, methodology, writing – original draft. Peng Wang: writing – review and editing, methodology, conceptualization, methodology, software. Nan Li: funding acquisition, supervision. Yan Gao: project administration, funding acquisition.

## Funding

The authors have nothing to report.

## Ethics Statement

The study was approved by the Animal Medical Research Ethics Committee Branch of the General Hospital of Northern Theater Command (Approval Number: 2024‐39).

## Conflicts of Interest

The authors declare no conflicts of interest.

## Supporting information


**Data S1:** Methods.

## Data Availability

The original contributions presented in the study are included in the article; further inquiries can be directed to the corresponding authors.
