# Supplementary material for: PPARγ‐Dependent Pioglitazone Treatment Suppresses Neuroinflammation and Improves Cognition in Sepsis‐Associated Encephalopathy in Mice
Source: CNS Neurosci Ther. 2026 Aug 30;32(9):e71103. doi: 10.1002/cns.71103 (PMC13527159; doi:10.1002/cns.71103)
Supplement: Supplementary file 1 [file CNS-32-e71103-s001.doc]

## Supplementary Materials: Methods

### Supplementary Method 1. Experimental materials and instruments

#### Reagents

Pioglitazone (Sigma–Aldrich, USA); GW9662 (Kebio Biotechnology, Nanjing, China); isoflurane (Hebei Yipin Pharmaceutical, China); chloral hydrate (Beijing Chemical Works, China); general-purpose tissue fixative (Servicebio, Wuhan, China). DMSO, lysis buffer, protease inhibitor cocktail, BCA protein assay kit, polyacrylamide gel reagents, SDS, Western ECL substrate and TEMED (Solarbio, Beijing, China). 5× dual-colour loading buffer (Fude Bio, Hangzhou, China). Dual-colour prestained protein marker (Yeasen, Shanghai, China). Methanol (Sinopharm Chemical Reagent, China). Skim milk powder (Yili Group, Inner Mongolia, China). Phosphate-buffered saline (PBS) powder packets (pH 7.4) (Eyou Bio, China). Primary antibodies against TLR4, NF-κB and β-actin (Proteintech, USA); caspase-3, Bax and Bcl-2 (Abcam, USA); Claudin-5 (Invitrogen, USA). HRP-conjugated secondary antibodies (goat anti-rabbit IgG; goat anti-mouse IgG) (Abcam, USA). ELISA kits for TNF-α, IL-6, IL-1β and IL-10 (Xitang Bio, Shanghai, China). 0.9% sodium chloride injection (100 ml and 500 ml) (Huarun Pharmaceutical, China).

#### Equipment

Isoflurane anaesthesia system for mice (ASA, UK); ice maker (IMS-40; Xueke Electric Appliance, Changshu, China); forced-air drying oven (Jinghong, Shanghai, China); refrigerated high-speed centrifuge (Sigma, Germany); ultrasonic cell disruptor (Thermo Fisher Scientific, China); thermostatic magnetic stirrer (Jiangnan Instrument Factory, Jintan, Changzhou, China); 4 °C refrigerator (Haier BCD-236DB, China). Vertical electrophoresis apparatus, power supply, transfer tank and microplate reader (Bio-Rad, USA). Induction cooker (Midea, China). Light microscope (Olympus, Japan). Analytical balance (Jingke Tianmei, Shanghai, China). Automatic tissue processor, paraffin embedding station and microtome (Leica, Germany). Vortex mixer (Qilinbeier, Haimen, China). Vertical pressure cooker/autoclave (Boxun, Shanghai, China). Temperature-controlled shaking incubator (ONUO, Tianjin, China). Chemiluminescence/fluorescence imaging system (Tanon, Shanghai, China). −80 °C ultra-low temperature freezer (Haier Special Appliances, Qingdao, China).

****Quantification of inflammatory cytokines in hippocampal tissue****

Inflammatory cytokine levels in hippocampal tissue were measured using commercially available mouse ELISA kits for TNF-α, IL-1β, IL-6, and IL-10 (Xitang Bio, Shanghai, China) according to the manufacturer’s instructions. Briefly, frozen hippocampal tissues were thawed on ice and homogenized in lysis buffer. The homogenates were incubated on ice and then centrifuged at 12,000 r.p.m. for 20 min at 4°C. The supernatants were collected for cytokine detection, and total protein concentration was measured using a BCA protein assay kit. Samples were diluted before ELISA measurement. Standards and diluted samples were added to the ELISA plate at 100 μl per well, and absorbance was measured using a spectrophotometric microplate reader. Cytokine concentrations were calculated from the corresponding standard curves and expressed after normalization to total protein concentration.

### Supplementary Method 2. Induction of sepsis by caecal ligation and puncture

Mice were anaesthetised with isoflurane using an anaesthesia system set to an oxygen flow rate of 200–300 ml min⁻¹, with 3–4% isoflurane for induction and 1–1.5% for maintenance. After the loss of pedal and tail pinch reflexes, mice were placed supine on a 37 °C heated surgical platform. The abdomen was shaved and disinfected with povidone–iodine three times, alternating clockwise and counter-clockwise application. After air-drying, a sterile drape was applied. A ~1 cm midline incision was made in the lower abdomen, and the caecum was exteriorised toward the right lower quadrant. The caecum was ligated with 6-0 suture at a point >1 cm from the distal tip. Using a 19-gauge needle, two punctures were made on the same side of the caecum; a small amount of faecal material was gently extruded, and the caecum was returned to the peritoneal cavity. The abdomen was closed with 6-0 suture and the incision was disinfected. After recovery from anaesthesia, mice received fluid resuscitation with 0.9% NaCl (30 ml kg⁻¹, subcutaneous). The CLP procedure was performed with reference to established experimental sepsis protocols^[S1]^.

### Supplementary Method 3. Behavioural testing

#### Open field test

The open field test was used to assess locomotor activity and exploratory behaviour, based on the conflict between novelty-driven exploration and innate avoidance of open areas. The arena consisted of an uncovered plastic box (50 × 50 × 40 cm) assembled from medical-grade acrylic panels. A Sony Super HAD CCD camera was mounted ~1 m above the arena and connected to a computer running SuperMaze (Shanghai Xinruan Information Technology Co., Ltd.). The arena was divided into 25 equal squares (5 × 5). Each mouse was placed in the centre and allowed to explore freely for 5 min; noise and other disturbances were avoided throughout testing. Data were analysed using the following formula:
Percentage of time (or entries) in the centre (%) = centre time (or centre entries) / total time (or total entries) × 100 ^[S2]^.

#### Elevated plus maze

The elevated plus maze was used to evaluate anxiety-like behaviour arising from the conflict between fear of open, elevated arms and exploration of a novel environment. The apparatus comprised a plus-shaped acrylic maze elevated ~1 m above the floor, with two open arms (35 × 5 cm) and two closed arms (35 × 5 × 15 cm). A Sony Super HAD CCD camera was positioned ~1 m above the maze to record trajectories. Each mouse was placed in the central zone facing an open arm and allowed to explore for 5 min. Data were analysed as:
Percentage of open-arm entries (or time) (%) = open-arm entries (or open-arm time) / total entries (or total time) × 100 ^[S3]^.

#### Novel object recognition

Novel object recognition was used to assess spontaneous exploration and recognition memory in a minimally stressful, ethologically relevant setting. The arena (40 × 40 × 40 cm) was constructed from medical-grade acrylic panels. A Sony Super HAD CCD camera was mounted ~1 m above the arena, and SuperMaze was used for acquisition and tracking. Two red wooden cubes (3 cm edge length) and one yellow cylinder (height 3 cm; diameter ~3 cm) served as objects.

Training phase: two identical cubes were placed in two corners, each 8 cm from the adjacent walls. The mouse was placed in the centre (oriented away from the objects), ensuring equal initial distance to both objects, and allowed to explore freely for 10 min.
Test phase: after a 1 h interval, one cube was replaced with the cylinder. The mouse was reintroduced as above, and exploration was recorded for 5 min.
Data were analysed using:
Exploration Index (%) = exploration time of either cube during training / total exploration time during training × 100;
Discrimination Index (%) = exploration time of the novel object during testing / total exploration time during testing × 100 ^[S4,S5]^.

#### Y-maze task

The Y-maze task was used to evaluate spatial learning and memory. The apparatus consisted of three arms (35 × 5 × 10 cm) arranged radially at 120° to form a “Y” shape. A Sony Super HAD CCD camera was placed ~1 m above the maze for tracking, and SuperMaze was used for data recording. Arms were designated as the correct arm, incorrect arm and start arm.

Mice were food-restricted 12 h before training (1–2 g food per mouse). On days 1–2, dark chocolate rewards were placed in all three arms, and mice underwent two training sessions per day. From days 3–6, rewards were placed only in the correct arm, and mice received one training session per day. On day 7, rewards were removed, and mouse trajectories were recorded for 5 min. Data were analysed as:
Percentage of correct-arm entries (or time) (%) = correct-arm entries (or correct-arm time) / total entries (or total time) × 100 ^[S6]^.

### Supplementary Method 4. Quantification of inflammatory cytokines in hippocampal tissue

Frozen hippocampal samples stored at −80 °C were thawed on ice. Each sample was homogenised in 70 μl lysis buffer using a pointed glass rod in a 1.5 ml microcentrifuge tube to generate a suspension. Homogenates were incubated on crushed ice for ~20 min and centrifuged at 12,000 r.p.m. for 20 min at 4 °C. The supernatant was collected, and total protein concentration was determined by BCA assay; samples were then diluted 1:20. ELISA kits for TNF-α, IL-1β, IL-6 and IL-10 were used according to the manufacturer’s instructions. Briefly, 100 μl of standards or diluted samples was added per well, and absorbance was read using a spectrophotometric plate reader. Cytokine concentrations were calculated and reported as pg ml⁻¹.

### Supplementary Method 5. Immunoblotting

Hippocampal samples were processed as above to obtain clarified supernatants. Protein concentration was measured by BCA assay. For BCA quantification, samples were loaded into a 96-well plate and sequentially mixed with lysis buffer, sample and working reagent as specified by the kit; plates were incubated at 37 °C for 30 min, and optical density values were measured to derive protein concentrations. Samples were normalised and prepared at 2 μg μl⁻¹ (100 μl per sample).

Proteins were separated on 10% SDS–PAGE gels. Samples were loaded in the order: Sham, Sham + Pio, CLP, CLP + Pio, CLP + GW9662 and CLP + Pio + GW9662 (20 μl per lane). Electrophoresis was run for ~1.5 h, followed by membrane transfer for ~2.5 h. Membranes were blocked in skim milk for4h, then incubated with primary antibodies with shaking at80r.p.m. for1h and subsequently overnight. The antibody types and dilution ratios used for western blotting are listed in Supplementary TableS1. Membranes were washed in PBST three times(10min each), incubated with the corresponding secondary antibodies for4h, and developed using ECL substrate.Chemiluminescent signals were captured and band intensities quantified using the Tanon imaging system.The immunoblotting procedure was performed with reference to established western blotting protocols^[S7,S8]^.

#### Supplementary Table S1. Antibody types and dilution ratios in western blot experiments

| **Antibody** | **Antibody type** | **Dilution ratio** |
| --- | --- | --- |
| β-actin | Mouse monoclonal IgG | 1:2000 |
| TLR4 | Mouse monoclonal IgG | 1:500 |
| Bcl-2 | Mouse monoclonal IgG | 1:500 |
| Bax | Rabbit monoclonal IgG | 1:500 |
| Claudin-5 | Rabbit monoclonal IgG | 1:500 |
| Caspase 3 | Rabbit monoclonal IgG | 1:200 |
| NF-κB | Rabbit monoclonal IgG | 1:500 |
| Secondary antibody | Goat anti-rabbit IgG-HRP | 1:5000 |
| Secondary antibody | Goat anti-mouse IgG-HRP | 1:5000 |

### Supplementary Method 6. Immunofluorescence

#### Blood–brain barrier permeability (Evans blue)

Evans blue was prepared as a 2% solution by dissolving 0.2 g Evans blue in 10 ml 0.9% NaCl. Mice were restrained using a tail-vein injector, the tail was exposed, and 2% Evans blue was administered via the tail vein (3 mg kg⁻¹; 0.3 ml per mouse). Two hours later, the thoracic cavity was opened, the heart was exposed, and the right atrial appendage was incised. Mice were transcardially perfused via the left ventricle with 20 ml 0.9% NaCl until the effluent from the right atrium became clear, followed by perfusion with 20 ml 4% paraformaldehyde (PFA) using the same approach. Brains were collected and stored at 4 °C, then cryoprotected sequentially in 20% and 30% sucrose. Tissue was sectioned into ~30 μm cryosections. Sections were permeabilised with 0.1% Triton X-100 at room temperature for 10 min, washed three times in PBS (5 min each), and counterstained with DAPI for 5 min in the dark. After three PBS washes (5 min each), sections were mounted with glycerol and coverslipped. Fluorescence images were acquired using a fluorescence microscope. Evans blue extravasation was used as an indicator of blood–brain barrier permeability according to previously described methods^[S9,S10]^.

#### TUNEL staining

Brains fixed in 4% PFA were paraffin-embedded and sectioned at ~30 μm. After deparaffinisation and rehydration, TUNEL staining was performed according to the manufacturer’s instructions. Slides were placed in a light-protected box and treated sequentially with proteinase K and 5% goat serum. rTdT buffer was applied to the sections, followed by incubation at 37 °C for 1 h. Sections were washed three times in PBS (5 min each), counterstained with DAPI for 5 min in the dark, washed again three times in PBS (5 min each), and mounted with glycerol. Images were captured using a fluorescence microscope. TUNEL staining was performed with reference to the established method for detecting DNA fragmentation in situ^[S11]^.

#### BrdU/NeuN immunofluorescence

BrdU powder stored at −20 °C was dissolved in 0.9% NaCl to a final concentration of 5 mg ml⁻¹. One day before tissue collection, three mice per group were randomly selected and injected intraperitoneally with BrdU three times per day; tissues were collected 8 h after the final injection. Mice were perfused transcardially as described above (20 ml 0.9% NaCl followed by 20 ml 4% PFA). Brains were post-fixed at 4 °C, dehydrated, embedded, and sectioned at ~30 μm. After deparaffinisation and rehydration, slides were placed in a light-protected box and treated sequentially with proteinase K and 5% goat serum, then incubated with anti-BrdU antibody (1:200) for 48 h and anti-NeuN antibody (1:200) for 4 h. Nuclei were counterstained with DAPI for 5 min. Sections were mounted with glycerol and coverslipped, and fluorescence images were acquired using a fluorescence microscope. BrdU incorporation was used to label DNA replication based on previously described methods^[S12]^.

**Supplementary References**

[S1] Rittirsch D, Huber-Lang MS, Flierl MA, Ward PA. Immunodesign of experimental sepsis by cecal ligation and puncture. Nat Protoc. 2009;4(1):31-6. doi: 10.1038/nprot.2008.214. PMID: 19131954; PMCID: PMC2754226.

[S2] Kraeuter AK, Guest PC, Sarnyai Z. The Open Field Test for Measuring Locomotor Activity and Anxiety-Like Behavior. Methods Mol Biol. 2019;1916:99-103. doi: 10.1007/978-1-4939-8994-2_9. PMID: 30535687.

[S3] Lister RG. The use of a plus-maze to measure anxiety in the mouse. Psychopharmacology (Berl). 1987;92(2):180-5. doi: 10.1007/BF00177912. PMID: 3110839.

[S4] Ennaceur A, Delacour J. A new one-trial test for neurobiological studies of memory in rats. 1: Behavioral data. Behav Brain Res. 1988 Nov 1;31(1):47-59. doi: 10.1016/0166-4328(88)90157-x. PMID: 3228475.

[S5] Antunes M, Biala G. The novel object recognition memory: neurobiology, test procedure, and its modifications. Cogn Process. 2012 May;13(2):93-110. doi: 10.1007/s10339-011-0430-z. Epub 2011 Dec 9. PMID: 22160349; PMCID: PMC3332351.

[S6] Kraeuter AK, Guest PC, Sarnyai Z. The Y-Maze for Assessment of Spatial Working and Reference Memory in Mice. Methods Mol Biol. 2019;1916:105-111. doi: 10.1007/978-1-4939-8994-2_10. PMID: 30535688.

[S7] Towbin H, Staehelin T, Gordon J. Electrophoretic transfer of proteins from polyacrylamide gels to nitrocellulose sheets: procedure and some applications. Proc Natl Acad Sci U S A. 1979 Sep;76(9):4350-4. doi: 10.1073/pnas.76.9.4350. PMID: 388439; PMCID: PMC411572.

[S8] Mahmood T, Yang PC. Western blot: technique, theory, and trouble shooting. N Am J Med Sci. 2012 Sep;4(9):429-34. doi: 10.4103/1947-2714.100998. PMID: 23050259; PMCID: PMC3456489.

[S9] Waithe OY, Peng X, Childs EW, Tharakan B. Measurement of Blood-Brain Barrier Hyperpermeability Using Evans Blue Extravasation Assay. Methods Mol Biol. 2024;2711:177-184. doi: 10.1007/978-1-0716-3429-5_14. PMID: 37776457.

[S10] Saunders NR, Dziegielewska KM, Møllgård K, Habgood MD. Markers for blood-brain barrier integrity: how appropriate is Evans blue in the twenty-first century and what are the alternatives? Front Neurosci. 2015 Oct 29;9:385. doi: 10.3389/fnins.2015.00385. PMID: 26578854; PMCID: PMC4624851.

[S11]Gavrieli Y, Sherman Y, Ben-Sasson SA. Identification of programmed cell death in situ via specific labeling of nuclear DNA fragmentation. J Cell Biol. 1992 Nov;119(3):493-501. doi: 10.1083/jcb.119.3.493. PMID: 1400587; PMCID: PMC2289665.

[S12] Gratzner HG. Monoclonal antibody to 5-bromo- and 5-iododeoxyuridine: A new reagent for detection of DNA replication. Science. 1982 Oct 29;218(4571):474-5. doi: 10.1126/science.7123245. PMID: 7123245.
